# Another New Medical Law

**Published:** 1890-11

**Authors:** 


### ANOTHER NEW MEDICAL LAW.

We call the attention of our readers in this State to the following circular letter, addressed to the medical profession, which explains itself :

Buffalo, N. Y., June 2, 1890.

*Dear Doctor :* — The committee of the Medical Society of the State of New York for the Prevention of Blindness enclose to you herewith a copy of a law passed by the Legislature during the last session. By thus calling the attention of certain members of the profession, of examiners of midwives, and of midwives themselves, to the existence of such a law, it is hoped that its purpose may be better fulfilled. The committee would urge the desirability of promptly reporting cases of its violation to the County District

Attorney, or other proper legal authorities, either directly, or through the County Medical Societies. And, with a view to even more stringent regulations in the future, the chairman of the committee would be obliged for information regarding any cases of conviction under this law.

Very sincerely yours,

LUCIEN HOWE, *Chairman*,
183 Delaware avenue, Buffalo.

HENRY D. NOYES,
233 Madison avenue, New York.

E. V. STODDARD,
68 South Washington street, Rochester.

AN ACT FOR THE PREVENTION OF BLINDNESS IN INFANTS.

*The People of the State of New York, Represented in Senate and Assembly, do enact as follows:*

SECTION 1. Should any midwife or nurse having charge of an infant in this State, notice that one or both eyes of such infant are inflamed or reddened, at any time within two weeks after its birth, it shall be the duty of such midwife or nurse so having charge of such infant, to report the fact in writing, within six hours, to the health officer, or some legally qualified practitioner of medicine, of the city, town, or district in which the parents of the infant reside.

§ 2. Any failure to comply with the provisions of this act shall be punished by a fine not to exceed one hundred dollars, or imprisonment not to exceed six months, or both.

§ 3. This act shall take effect on the first of September, eighteen hundred and ninety.

